# Prevalence of Intestinal Parasitic Infection in Cancer, Organ Transplant and Primary Immunodeficiency Patients in Tehran, Iran

**DOI:** 10.31557/APJCP.2019.20.2.495

**Published:** 2019

**Authors:** Abdoulreza Esteghamati, Khadijeh Khanaliha, Farah Bokharaei-Salim, Shirin Sayyahfar, Masoomeh Ghaderipour

**Affiliations:** 1 *Research Center of Pediatric Infectious Diseases, Institute of Immunology and Infectious Diseases, *; 2 *Departments of Virology, School of Medicine, Iran University of Medical Sciences, Tehran, Iran. *

**Keywords:** Immunodeficiency, transplant, cancer, opportunistic, parasite, infection

## Abstract

**Background:**

Intestinal parasitic infection in immunodeficient patients especially those with impaired cellular immunity, like neoplasia, renal or heart transplant needs careful consideration. The objective of this study is to evaluate the prevalence of intestinal parasites in different group of patients including cancer patients; organ transplants recipients, and primary immunodeficiency patients.

**Methods:**

Stool samples from 190 patients including 80 patients with Primary Immunodeficiency, 85 cancer patients and 25 organ transplant recipients were collected; a direct examination with Phosphate buffered saline (PBS) and formalin ether concentration was performed. The DNA was extracted from parasitologically confirmed patients and nested PCR and sequencing was performed and new obtained sequences of *Cryptosporidium parvum *and *Enterocytozoon bieneusi* were compared with deposited ones.

**Results:**

In general, the prevalence of parasites was 26/80 (32.5%) in primary immunodeficiency, 22/85(25.9%) in cancer group, and 7/25 (28%) in organ transplant. The prevalence of intestinal parasitic infections in primary immunodeficiency patients were *Blastocystis hominis *13 (16.2%), *Giardia lamblia* 10 (12.5%), *Cryptosporidium* 1(1.2%), *Chilomastix mesnilii* 1 (1.2%), *Dientamoeba fragilis *1(1.2%). Of 25 organ transplants, 6 (24%) *Cryptosporidium sp* were found, all of which were confirmed as *Cryptosporidium parvum *and one case of Microspora in a heart transplant recipient was confirmed as *Enterocytozoon bieneusi *by PCR sequencing. The predominant intestinal parasitic infection in cancer patients was 19 (22.3%) *Blastocystis hominis *followed by two (2.3%)* Giardia lamblia* and one* Dientamoeba fragilis* 1 (1.1%).

**Conclusion:**

The high rate of infection with *Blastocystis hominis *was found in cancer patients especially colorectal cancer patients, so careful consideration should be given by physicians. *Cryptosporidium sp* was found to be the major cause of parasitic intestinal infection in patients with organ transplant compared to primary immunodeficiency patients; so transplant recipients undergoing immunosuppressive therapy should be considered as a risk group for acquiring microsporidiosis and *Cryptosporidium* infection.

## Introduction

In developing countries, intestinal parasitic infection in immunodeficient patients including, organ transplant recipients, cancer and common variable immunodeficiency patients needs careful consideration. Opportunistic infections are the cause of diarrhea in patients with HIV/AIDS (Khanaliha et al., 2015) and patients under some kind of immunosuppression drugs (Rasti et al., 2017).

Nowadays the increasing use of transplants (kidney, bone marrow, liver, heart, etc.) and the use of new immunosuppressive drugs in patients with cancer and primary autoimmune diseases are observed. (Ferreira and Borges, 2002).

The most frequently reported malignancies were stomach cancer in males and breast cancer in females in Tehran, Iran during 1998 – 2001 (Mohagheghi et al., 2009). Colorectal cancer (CRC) is one the most common cancers worldwide. This cancer is the third most common cancer among Iranian men. According to the results of a study, the incidence of colorectal cancer has increased in Iran during 2003 and 2008 (Rafiemanesh et al., 2016).

It is demonstrated that oxidative stress plays a critical role in the molecular mechanisms of colorectal cancer (CRC) and is shown to be associated with *Blastocystis sp*, that is the most common intestinal protozoa (Kumarasamy et al., 2017). The pathogenicity of *Blastocystis sp.* is associated with some disease like acquired immune deficiency syndrome (AIDS) (Fisseha et al., 2017) and colorectal cancer (CRC) (Chandramathi et al., 2014). *Blastocystis sp *in CRC patients during chemotherapy treatment displays opportunistic nature of* Blastocystis sp *in these patients(Chandramathi et al., 2012). 

The second most prevalent primary immunodeficiency is common variable immunodeficiency (CVID) disease. CVID is a combination of humoral and cell-mediated deficiency. It is defined as hypogammaglobulinemia associated with IgG and IgA immunoglobulin deficit whereas the immunoglobulin M (IgM) concentration can be normal or low (Chapel and Cunningham-Rundles, 2009). An increased risk of malignancy especially lymphoma and gastric cancer is associated with CVID and malignancy is diagnosed as one of the five clinical CVID phenotypes (Chapel et al., 2008; Mortaz et al., 2016).

Although intravenous immunoglobulin therapy was performed on CVID patients because of the defects of T-cell-mediated, the gastrointestinal disorders were observed (Kalha and Sellin, 2004).The intestinal parasitic infection like *Giardia lamblia*, *Cryptosporidium parvum * are common in patients with primary immunodeficiencies (PIDs) patients group (Aguilar et al., 2014).

Parasitic infections rate in organ transplant recipients is unknown, however few patients are symptomatic (Thom and Forrest, 2006).Only 5% of known human pathogenic parasitic infections which have serious morbidity have been reported in transplant recipients (Barsoum, 2004). The transplant clinicians should be highly suspicious of parasitic infections as an important transmission threat (Barsoum, 2004). 

*Cryptosporidium* is a coccidian intestinal protozoan, which is often found in the human intestine that can cause severe diarrhea in the immunocompromised patient. It is an infamous infection in ileum transplants (Pozio et al., 2004), but has also been reported as a recrudescence disease in recipients of liver (Campos et al., 2000), kidney (Minz et al., 2004) , and bone marrow transplants (Müller et al., 2004).*Cryptosporidium* should be differentiated from other protozoa, Isospora belli and Cyclospora cayetanensis in immunocompromised patients (Verweij and Stensvold, 2014).

The genus *Cryptosporidium* consist of over 20 species which species like *C. parvum* from *C. hominis* are indistinguishable morphologically (Peralta et al., 2016). *Cryptosporidium* infection in both immunocompromised and immunocompetent individuals may be found by different species and genotypes (Cama et al., 2007). 

Microsporidia are intracellular spore-forming protozoa that are present in the environment and can infect the intestine of insects, birds, and mammals. The diagnosis was made by staining and molecular method (Agholi et al., 2013a; Khanaliha et al., 2014). Enterocytozoon bieneusi in patients with HIV disease was reported in several studies (Agholi et al., 2013a). 

The aim of this study is to evaluate the prevalence of intestinal parasites in different groups of patients including organ transplants recipients, cancer group and primary immunodeficiency referred to Milad hospital and three general hospitals namely Ali-Asghar, Rasoul-e-Akram hospital, and Rajaie Heart Hospital associated with Iran University of Medical sciences, Tehran, Iran.

## Materials and Methods

This cross-sectional study was performed between July 2016 and November of 2017 including 80 patients with Primary Immunodeficiency, 85 cancer patients and 25 organ transplant recipients who were referred to Milad hospital and three general hospitals namely Ali-Asghar, Rasoul-e-Akram hospitals and Rajaie Heart Hospital associated with Iran University of Medical Sciences, Tehran, Iran. 

*Stool examination*

Stool samples were collected from 190 patients referred to hospitals associated with Iran University of Medical Sciences and transferred to Research Center of Pediatric Infectious Diseases, Institute of Immunology and Infectious Diseases, Iran University of Medical Sciences. Furthermore, they were examined by direct examination with PBS and then formalin ether concentration was performed and finally examined under microscope with 100x and 400x as final magnification. The modified acid fast staining method was applied for the detection of coccidia parasites infection. The smears were prepared and stained by the modified Ziehl–Neelsen stain. The aniline blue staining method was used to detect spores of microsporidia as described before (Ghaderipour et al., 2017).

*DNA extraction*

All of samples were positive for *Cryptosporidium* and microspora by Ziehl–Neelsen technique and aniline blue staining method respectively were used in PCR. DNA extraction was performed by (Roche Diagnostics GmbH, Mannheim, Germany) according to the manufacturer’s instructions

*Cryptosporidium*

The nested PCR was performed and an 837 bp fragment amplified of the 18S rRNA gene. The primers were used for nested PCR primers described by Santin et, al (Santín et al., 2006).

5′-TTCTAGAGCTAATACATGCG-3′ and 5′-CCCATTTCCTTCGAAACAGGA-3′ for primary PCR and 5′-GGAAGGGTTGTATTT-ATTAGATAAAG-3′ and 5′-AAGGAGTAAGGAACAACCTCCA-3′ for secondary PCR.

PCR was performed in a 25 µL mixture containing the template (3 µL of DNA), 2.5 U of Taq DNA polymerase, 2.5 µL of 10x PCR buffer, 20 pmol of each primer, 100 µmol dNTPs, and 0.15 mmol MgCl_2_. After an initial start at 94 ^oC^ for 3 min, each of cycles consisted of 94 ^oC^ for 45 s, 59 ^oC^ for 45 s and72 ^oC^ for 1 min for 35 cycles and ends with 72^oC^ for 7 min. The second round was performed by the cycling program consisting of 40 cycles 94 ^oC^ for 30 s, 58 ^oC^ for 90 s, 72 ^oC^ for 20 min, with an initial at start 94 ^oC^ for 3 min and ended with 72 ^oC^ for 7 min. Finally, PCR products were electrophoresed on 1 % agarose gel. The positive control was used in each step of PCR and distilled water was used as a negative control.

*Enterocytozoon bieneusi*

The nested PCR was carried out using *E. bieneusi *specific primers that amplified the ITS region as well as a portion of the flanking large and small subunit ribosomal RNA genes (Santín et al., 2006)

The outer primers were EBITS3 (5′-GGTCATAGGGATGAAGAG-3′) and EBITS4 (5’-TTCGAGTTCTTTCGCGCTC-3’). The inner primers were EBITS1 (5’-GCTCTGAATATCTATGGCT-3′) and EBITS2.4 (5′-ATCGCCGACGGATCCAAGTG-3′). Finally, these reactions produced a fragment of 389 bp

The reaction mixture (25 µL) contained 2.5 µL of 10x PCR buffer 0.15 mmol MgCl2, 100 µmol dNTPs, 20 pmol of each primer, 2.5 U of Taq. The PCR started with denaturation at 94 ^oC^ for 5 min, 35 cycles of amplification (denaturation at 94 ^oC^ for 30 s, annealing at 57^oC^ for 30 s, and elongation at 72^oC^ for 40 s) and then was followed by a final extension at 72 ^oC^ for 10 min. The annealing temperature for the second round of PCR was 55 ^oC^ and other condition was identical with first round of PCR (Santín et al., 2006).

*Ethical issues*

The present study was approved by the Ethics Committee of Iran University of Medical Sciences in accordance with the Helsinki Declaration and Guidelines. It also should be mentioned that informed consent was obtained from all patients for the present study. 

Sequencing: The second round of PCR was done using inner primers and PCR products were purified by the High Pure PCR Product Purification Kit (Roche Diagnostic, Mannheim, Germany) and were used for direct sequencing using the dye termination method and an ABI 3730xl sequencer (Khanaliha et al., 2017).

The new sequences of *Cryptosporidium* and *E. bieneusi *were deposited in GenBank database and compared with deposited sequences by BLAST analysis. 

Statistical analysis: Analysis was done using SPSS version 18 (Chicago, IL, USA) and Chi-square test was used to analyze statistical relationship. A statistically significant P value of < 0.05 was accepted.

## Results

Overall 190 patient consist of 109 (57.4%) males and 81 (42.6%) females including 80 CVID and Bruton agammaglobulinemia, 85 cancers group patients: lymphoma cancer, lymphosarcoma, hepatoblastoma, lung and colon cancer and 25 organ transplant including liver, kidney and heart were evaluated for parasitic infection 

Out of 80 CVID positive patients, 41 (51.2%) were male and 39 (48.8%) were female and among 85 cancer group patients, 48(56.5%) were male and 37 (43.5%) were female and of 25 organ transplant recipients 19 (76%) were males and 6 (24%) were females. The patient age was between 5-65 years old with the mean age 34 year.

In general, the prevalence of parasites were 26/80 (32.5%) in primary immunodeficiency, 22/85 (25.9%) in cancer group, 7/25 (28%) in organ transplant. The rate of infection was higher in male (31.2%) than female (18.9%) cancer patients and also in male (31.5%) and female (16.6%) OTR patients, but differences were not statistically significant (P value = 0.1) (P value = 0.4). The rate of infection was (31.7%) in male and (33.3%) in female CVID patients and differences were not statistically significant (P value = 0.4).

The prevalence of intestinal parasitic infection in primary immunodeficiency patients was *Blastocystis hominis *13 (16.2%), *Giardia lamblia* 10 (12.5%), *Cryptosporidium* 1 (1.2%), *Chilomastix mesnilii* 1 (1.2%), *Dientamoeba fragilis* 1 (1.2%).

Among 85 cancers patients including 39 colorectal cancer and 46 cancers outside gastrointestinal tract (COGT) like lymphoma, lymphosarcoma, hepatoblastoma, lung cancer 19 (22.3%) *Blastocystis hominis *and two (2.3%) *Giardia lamblia* and one *Dientamoeba fragilis* 1(1.1%) were found. *Blastocystis hominis *were identified in 11/39 (28.2%) among CRC group and 8/46 (17.4%) among COGT patients. Although *Blastocystis hominis *was more common among CRC group patients than COGT ones, the difference between groups wasn’t statistically significant (P value= 0.17).

**Figure 1:**
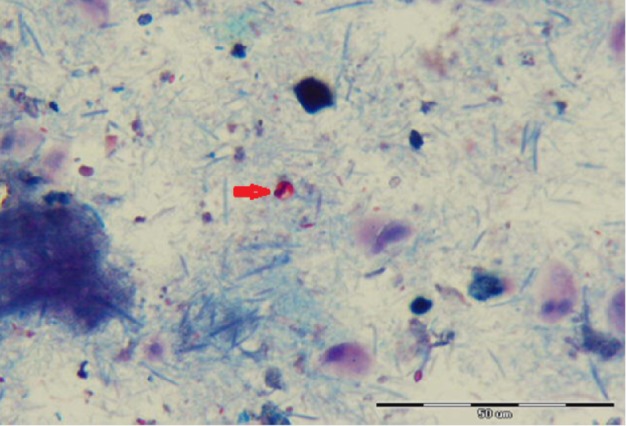
Appearance of *Cryptosporidium* Oocyst in Stool Samples Collected from Organ Transplant Recipients by Modified Acid Fast Staining Method, 1000x Magnification (Origin Picture)

**Figure 2:**
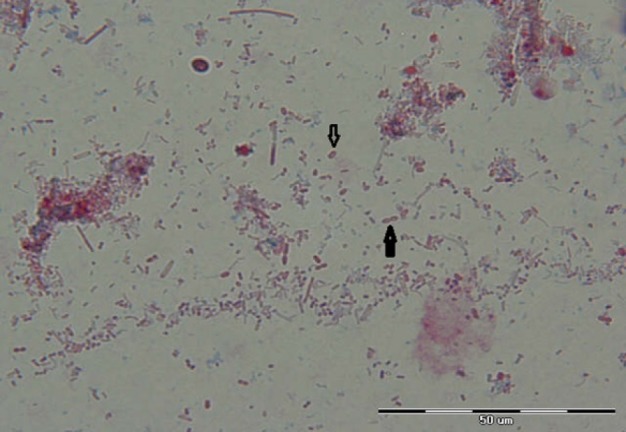
Appearance of *E. bieneusi* Spores in Stool Samples Collected from Heart Transplant Recipient by Aniline Blue Staining Method, 1,000x Magnification (Origin Picture)

**Table 1:** Prevalence of Intestinal Parasitic Infection among Different Groups of Patients

parasites	Primary immunodeficiency No: 80		Transplant recipients No:25		Cancers Patients No:85	
	Male	Female	Male	Female	Male	Female
*Blastocystis hominis*	6 (7.5%)	7 (8.75%)	0	0	12 (14.1%)	7 (8.2%)
*Giardia lamblia*	6 (7.5%)	4 (5%)	0	0	2 (2.3%)	0
*Cryptosporidium spp.	0	1 (1.25%)	5 (20%)	1 (4%)	0	0
*Chilomastix mesnilii*	0	1 (1.25%)	0	0	0	0
*Dientamoeba fragilis*	1 (1.25%)	0	0	0	1 (1.2%)	0
*Microspora	0	0	1 (4%)	0	0	0
Total	13 (16.25%)	13 (16.25%)	6 (24%)	1 (4%)	15 (17.6%)	7 (8.2%)

**Figure 3:**
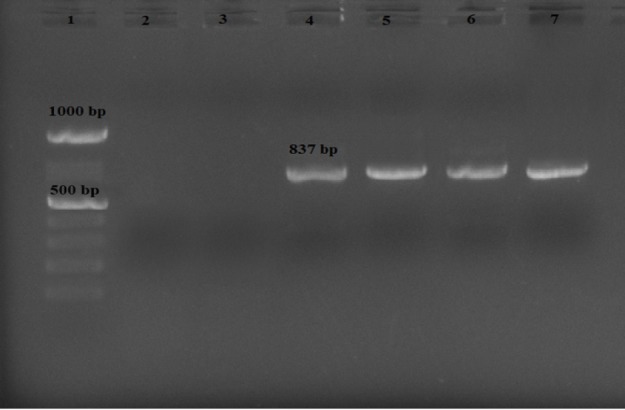
PCR Products of *Cryptosporidium* in Iranian Transplant Recipients. 1, DNA Ladder Marker; 2, Negative Control; 3, Negative Patient; 4, 5, 6, Positive Patients; 7, Positive Control

**Figure 4:**
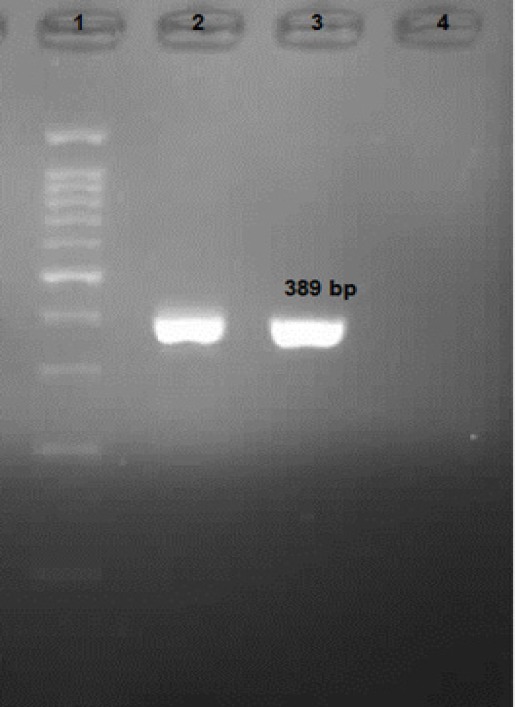
PCR Products of *E. bieneusi* in Iranian Transplant Recipient; 1, DNA Ladder Marker (100 bp); 2, Positive Control; 3, Positive *E. bieneusi* Patients (389 bp); 4, Negative Control

The results of this study show; 6 (24%) *Cryptosporidium sp* were found in 25 organ transplant recipients including (OTR) including liver, kidney and heart transplant. The oocyst of *Cryptosporidium* was found in three liver, two kidney and one heart transplant recipients and one additional *Cryptosporidium sp* was identified in primary immunodeficiency patients by acid fast staining method (Figure 1). One microspora 1 (4%) was found in a heart transplant recipient using aniline blue staining method (Figure 2). All of patients with cryptosporidiosis and microsporidia infection had diarrhea as a clinical manifestation. 

Microspora confirmed as *E. bieneusi *in a heart transplant recipients by PCR sequencing in this study. The results of prevalence of parasitic infection are summarized in Table 1.

There was no statistically significant difference between the prevalence of parasitic infection in cancer, CVID and OTR patients in this study (P value= 0.22; P value= 0.43), however, the difference between *Cryptosporidium* infection in OTR patients with CVID and OTR with cancer patients were statistically significant (P value= 0.0001)

*PCR and Sequencing*

The result of *Cryptosporidium* PCR is shown in Figure 3.The band of 837 bp fragment of the 18S rRNA gene related to *Cryptosporidium* was amplified from fecal specimens of patients by nested PCR protocol. All PCR positive samples were sequenced to identify the species. The obtained sequences were deposited and compared with other deposited sequences in the GenBank database by BLAST analysis. All of 7 patients infected with *Cryptosporidium* were also confirmed as *C. parvum* by PCR sequencing. The isolates had 100% similarity with the *C. parvum* genotype 1 (Gen Bank accession no. L16997).

The deposited sequences Gen Bank accession numbers for *Cryptosporidium* were: MH215510, MH215511, MH215512, MH215513, MH215514, MH215515, and MH215516 in this study.

Enterocytozoon bieneusi was identified in a heart transplant recipient by PCR sequencing. A band of 389 bp was observed in agarose gel electrophoresis of PCR product (Figure. 4). *E. bieneusi *isolate had 99% identity with G2-J9, 536 and CHN1 and genotype J in this study. The deposited sequences GenBank accession no for *E. bieneusi *was MH243443.

## Discussion

Parasitic infections are among the most important pathogens that can cause infections in immunocompromised individuals (Ferreira and Borges, 2002). Patients with impaired cellular immunity, like neoplasia, renal or heart transplant patients are at risk of protozoa infection (Ferreira and Borges, 2002).

In this study *Blastocystis hominis *with the prevalence of 19 (22.3%) was found as the most prevalent parasitic infection among cancers patients. *Blastocystis hominis *11/39 (28.2%) was detected more frequently in CRC group than COGT patients 8/46 (17.4%).

Prevalence of *Blastocystis sp *is relatively high in developing countries (Tan, 2008) and it was reported as the most common parasites in Iran (Mahni et al., 2016).

The result of a study demonstrated that *Blastocystis sp. *has a significant role in enhancing Azoxymethane (AOM) -induced carcinogenesis damage to the intestinal epithelium and oxidative damage were observed in rats infected with *Blastocystis sp *and the study proposed that *Blastocystis sp.* is a pathogen protozoa so it is necessary to screen cancer patients for this parasite (Kumarasamy et al., 2017). 

The prevalence of *Blastocystis sp *in CRC patients was 33.3% in comparison with 25% in cancers outside gastrointestinal tract (COGT) and 15% in non-cancer (NC) patients in a study and the result suggested a possible association between subtype-I of *Blastocystis hominis *and CRC, which could reveal an important role of *Blastocystis* on CRC condition (Mohamed et al., 2017).

Microsporidiosis has been reported in HIV positive patients but more recently, has been diagnosed in non–HIV-infected individuals (Agholi et al., 2013a; Ghaderipour et al., 2017).

Several studies have reported microsporidial infections in organ transplant recipients, however these infections have not been proved to be transplant-transmitted (Lanternier et al., 2009; Galván et al., 2011). The microsporidiosis have been reported in solid transplant recipients (Carlson et al., 2004), microsporidia were found in kidney recipients (Mohindra et al., 2002; Galván et al., 2011; Visvesvara et al., 2013), pancreas-kidney (Carlson et al., 2004) and heart transplants(Gumbo et al., 1999).

In the present study, *E. bieneusi *was found in a ten-year-old heart transplant patient who had chronic diarrhea and had received transplant for more than two years. Comparing the new sequence with the result of deposited ones, it was indicated that the *E. bieneusi *isolate had 99% identity with isolates G2-J9, 536 and CHN1 and genotype J in this study

Genotype CHN1 was previously detected in human and cattle as the most common genotype and genotype CHN2 was found only in human samples in a study (Zhang et al., 2011). 

In a study on among 44 liver transplant children in Shiraz 6.81% Enterocytozoon bieneusi (genotype D) and 11.36% *C. parvum* and *C. Meleagridis* were identified (Agholi et al., 2013b).

*Cryptosporidium* infection is more common in overcrowded societies with low degrees of sanitation (Brink et al., 2002) There are few studies on cryptosporidiosis in Iran (Azami et al., 2007). The previous reports indicated the prevalence of this infection in diarrhea patients which was 4.7% (Sulaiman et al., 2002) and in HIV-patients which was reported 1.5% (Guyot et al., 2001; Zali et al., 2004). 

*Giardia lamblia* and *Cryptosporidium parvum* are two parasites that may be found during Primary Immunodeficiency infection like agammaglobulinemia, CVID and Hyper-IgM syndrome with cellular defect, however, among the last cases, *Cryptosporidium parvum *is more common (Aguilar et al., 2014).

In the present study, *Blastocystis hominis *13 (16.2%), and *Giardia lamblia* 10 (12.5%) were the most prevalent parasites especially in primary immunodeficiency patients and one case *cryptosporidium parvum *1 (1.2%) was identified in a patient with CVID who suffered from chronic diarrhea.

*Blastocystis hominis *(4.2%) and *Giardia lamblia* (3.0%) were also reported as the most prevalent parasites in immunocompromised patients in the previous study (Rasti et al., 2017).

In a study which was performed in Turkey, the most common parasites diagnosed were *Cryptosporidium spp*, *Giardia spp* and *Blastocystis spp*, among CVID patients with clinical manifestation especially diarrhea (Uysal et al., 2016) . 

The most common *Cryptosporidium* infection was found more in organ transplant patients than cancer or patients with primary Immunodeficiency in the present study. All organ transplant recipients had received transplant for more than 1 year. Overall, 7 cases of *Cryptosporidium* identified that six of them were found in organ transplant recipients including liver, kidney and heart transplant recipients and one additional *Cryptosporidium* was detected in a CVID patient by acid fast staining. The result of PCR and sequencing show that all of them had 100% similarity with the *C. parvum* genotype 1, human genotype (GenBank accession no. L16997) and 99% similarity with *C. parvum* strain Human genotype (HFL2) (GenBank accession no. AF093491). 

In a study, four *Cryptosporidium* genotypes in HIV patients were identified: *C. parvum* genotype 1 (human), *C. parvum* genotype 2 (bovine), a genotype related to C. felis, and one genotype was similar to a *Cryptosporidium sp*. The corresponding sequences of the *C. parvum* genotype 1 SSU-rRNA were identical (GenBank accession number L16997) (Pieniazek et al., 1999).

A study which was carried out on immunocompromised Egyptian patients *Giardia lamblia* with prevalence of (10%) showed the most common parasite identified among patients. Other parasites were Cryptospotidium parvum (7%) Cyclospora cayetanensis (3%) and Microsporidia species (2%) (Baiomy et al., 2010).

The presence of *C parvum*, *C hominis*, and *C meleagridis* was confirmed in three patients with primary immunodeficiency by molecular method (Wolska-Kusnierz et al., 2007).

In a study, the 13 *Cryptosporidium* isolates from immunocompromised patients from Isfahan were evaluated and eight of them were infected with the *C. parvum* genotypes (GenBank accession no. L 16997) (Azami et al., 2007). *C. parvum* sequences, in the present study, had also 100% similarity with *C. parvum* genotypes (GenBank accession no. L 16997)

*C. parvum* isolates were detected among human and cattle and the L1 subgenotype was the most prevalent and was diagnosed in eight human cases out of nine and in all cattle cases, in a study (Meamar et al., 2007).It has been reported *C. parvum* cattle genotype are predominant among *C. parvum* isolates from human in some previous study (Azami et al., 2007; Meamar et al., 2007). 

Cancer patients especially CRC group had the highest rate of infection with *Blastocystis hominis *in this study, so screening of this protozoon for this group of patients with regard to the role of this parasite in oxidative damage to intestinal epithelium is recommended. Furthermore, *Blastocystis hominis *and *Giardia lamblia* were the most prevalent parasites in primary immunodeficiency and opportunistic infection like *Cryptosporidium* and *E. bieneusi *which were found more in OTR than primary immunodeficiency (CVID and Bruton), so these patients should be considered for opportunistic infection.

Patients with primary Immunodeficiency received azitromycine and were under prophylactic treatment in this study, so this may be a reason for reducing opportunistic infection like microspora and *cryptosporidium* in this group.

In general patients with impaired T-cell function especially to a CD4 counts of less than 50/mm^3^, whether it is inherited or acquired immunocompromised disease or patients under treatment depending on degrees of immune-suppression, are at higher risk of *Cryptosporidium* and microspora infection (Hunter and Nichols, 2002).

Overall, immunodeficient patients with clinical manifestation and impaired cellular immunity and under treatment of immune suppression should be considered for opportunistic parasitic infection.

In conclusion, the high rate of infection with *Blastocystis hominis *was found in cancer patients especially CRC patients, so careful consideration should be given by physicians. *Cryptosporidium parvum *was found to be the major cause of parasitic intestinal infection in patients with organ transplant compared to other CVID patients, so organ transplant recipients undergoing immunosuppressive treatment should be considered a risk group for acquiring microsporidia and *Cryptosporidium* infection. 

## Ethical considerations

This study was approved by the Ethics Committee of Iran University of Medical Sciences code number (IR.IUMS.REC1395-02-131-28109) in accordance in accordance with Helsinki Declaration and Guidelines. It also should be mentioned that informed consent has been obtained from all human participants.

## Sources of funding

This study was funded by Research Center of Pediatric Infectious Diseases, Iran University of Medical Sciences in Tehran, Iran with Grant number (95-02-131-28109). 

## Conflicts of interest

The authors declare that they have no conflicts of interest.
